# A Series of 35 Cutaneous Infections Caused by *Mycobacterium marinum* in Han Chinese Population

**DOI:** 10.1155/2023/5514275

**Published:** 2023-08-16

**Authors:** Wenjie Chen, Fangfang Bao, Qing Pan, Tingting Liu, Xiaotong Xue, Hong Liu, Furen Zhang

**Affiliations:** Shandong Provincial Hospital for Skin Diseases & Shandong Provincial Institute of Dermatology and Venereology, Shandong First Medical University and Shandong Academy of Medical Sciences, Jinan, China

## Abstract

Cutaneous *Mycobacterium marinum* infection is an increasingly infectious disease presenting unique diagnostic and therapeutic challenges. The aim of this study was to evaluate the differences in time to treatment among patients with different types of skin lesions and who were treated with single or multidrug therapies. In addition, the clinical characteristics of *M. marinum* infection were explored and the mechanism of the host immune responses was investigated. The electronic medical records of 35 patients with *M. marinum* infection were reviewed. The clinical characteristics, histopathological and laboratory data, and treatment outcomes were analyzed. Immunohistochemical analysis was performed to clarify the immune mechanisms induced by *M. marinum* infection in 9 patients and 5 healthy controls. Of the 35 patients, 25 (71.4%) had lesions with sporotrichoid patterns. The duration of patients with sporotrichoid lesions or treatment with multiple drugs was longer, although differences were not significant, possibly due to the small cohort. However, this trend was also observed in previous studies, making it worthy of further attention. Expression levels of cytokines (IFN-*γ*, IL-4, IL-9, and FOXP3) were significantly upregulated in the patient specimens, whereas there were no significant differences in IL-17 and IL-22 expression levels between the patient and control groups.

## 1. Introduction

*Mycobacterium marinum* is a slow-growing nontuberculous *Mycobacterium* (NTM) that was first isolated and identified from fish by Aronson in 1926 [1–3]. Later, in 1954, *M. marinum* was described as a human opportunistic pathogen by Collins et al. [3, 4]. Exposure to fish tanks and handling of fish are known risk factors for *M. marinum* infection, especially for individuals with minor skin trauma prior to contact with the infection source [5]. *M. marinum* infection usually presents as warty nodules or plaques, occasionally with a sporotrichoid pattern, which may progress to shallow crusting, ulceration, and eventual scar formation [6, 7]. Bacteremia resulting from *M*. *marinum* infection most commonly occurs in immunocompromised patients but is very rare [8]. Overall, cases of *M. marinum* infection are sporadic with an annual incidence of 0.27 cases per 100,000 population in the United States [9].

The diagnosis and treatment of *M. marinum* infection remain challenging, as the clinical presentation is often insidious and nonspecific. A diagnosis of *M*. *marinum* infection can be suspected by a history of contact with contaminated water or fish and confirmed by histologic evaluation and mycobacterial cultures. However, there is no consensus on an optimal antimicrobial regimen or treatment duration for *M. marinum* infection.

## 2. Materials and Methods

### 2.1. Study Approval and Patient Consent

The study protocol was approved by the Ethics Committee of Shandong Provincial Hospital of Dermatology (approval no. 20181218KYKT001) and conducted in accordance with the ethical principles for medical research involving human subjects described in the Declaration of Helsinki. Prior to inclusion in this study, written informed consent was obtained from all subjects.

### 2.2. Case and Data Selections

This study was conducted at the Department of Dermatology, Shandong Provincial Hospital of Dermatology (Jinan, Shandong, China). The study cohort consisted of 40 subjects, which included 35 patients with newly diagnosed cutaneous *M*. *marinum* infection and 5 uninfected healthy individuals as a control group. Normal control tissue specimens were obtained from the limbs of healthy individuals using a surgical scalpel under local anesthesia. The specimens were spindle-shaped and measured 1 × 0.5 × 0.5 cm in size. All *M. marinum* infections were confirmed by culture or quantitative real-time PCR (qPCR). The following data were collected from electronic medical records: patient sex and age, history of aquatic exposure, lesion site and clinical type, culture method, qPCR results, Ziehl–Neelsen staining results, type of antimicrobial therapy, and treatment outcome. The time to diagnosis was defined as the time from symptom onset to confirmation of *M. marinum* infection. The outcome was defined as cure with no sign of infection at the end of the follow-up period.

### 2.3. Histopathology and Laboratory Tests

All skin tissue specimens were fixed in a 10% buffered neutral formalin solution and cut into sections, which were separately stained with hematoxylin-eosin and fluorochrome staining for acid-fast bacilli (AFB). DNA was extracted from fresh tissue samples, and detection was performed concurrently using both qPCR and the *Mycobacterium marinum* PCR Kit (CAT#: 13-67830y, YaJi Biological, China).

The primer and probe sequences for qPCR analysis are listed in Supplementary Table 1. The qPCR reactions were carried out using a TaqMan Universal PCR Master Mix (Applied Biosystems, USA) on a StepOnePlus Real-Time PCR System (Applied Biosystems, USA). The cycling conditions were as follows: 50°C for 2 min and 95°C for 10 min, followed by 40 cycles of 95°C for 15 s and 60°C for 1 min. A positive control (*Mycobacterium* DNA) and a negative control (water) were included in each run. The threshold cycle (Ct) values were recorded and analyzed. A sample was considered positive for *M. marinum* infection if the Ct value was less than 35.

Skin biopsy specimens of all 35 patients were cultured on blood agar plates (Autobio Diagnostics Co., Ltd., Zhengzhou, China), Sabouraud dextrose agar (Hangzhou Binhe Microbial Reagent Co., Ltd., Hangzhou, China), and Lowenstein-Jensen culture medium (BaSO Diagnostics Inc., Zhuhai, China). The culture results were considered negative if no growth occurred within 6 weeks. The species of the cultured isolates were identified by PCR-based Sanger sequencing of 16S rRNA with the primer sequences listed in Supplementary Table 2.

All clinical isolates were tested for antimicrobial susceptibility using the Sensititre™ SLOMYCOI assay (Trek Diagnostic Systems Ltd., East Grinstead, West Sussex, England). Colonies were suspended in H_2_O, and the turbidity was measured against a 0.5 McFarland turbidity standard using a nephelometer. Then, 50 *μ*L of each suspension was mixed with 50 *μ*L of oleic acid albumin dextrose solution in separate wells of Sensititre™ MHB plates (Thermo Fisher Scientific, Waltham, MA, USA), which were sealed and incubated at 35°C in a non-CO_2_ incubator for 7 days. Based on the results of drug sensitivity testing, 26 patients with positive cultures received treatment, while 9 patients had negative culture results. Appropriate drugs were administered after confirmation of the pathogen by qPCR analysis.

Moreover, IHC analysis was performed on paraffin-embedded cutaneous biopsies from patients with confirmed *M. marinum* infection and uninfected controls. The expression levels of forkhead box P3 (FOXP3) (Abcam, Cambridge, MA, USA), interferon (IFN)-*γ* (Abcam, Cambridge, MA, USA), interleukin (IL)-4 (Proteintech, Wuhan, China), IL-9 (Proteintech, Wuhan, China), IL-17 (Proteintech, Wuhan, China), and IL-22 (Abcam, Cambridge, MA, USA) in the tissue specimens were measured and compared with the uninfected controls.

### 2.4. Data Analysis

Data analysis was performed using Microsoft Excel (Microsoft Corporation, Seattle, WA, USA), and statistical analyses were performed using R software v3.6.1 (https://cran.r-project.org/bin/windows/base/old/3.6.1/). Continuous variables are presented as the mean, median, and interquartile range (IQR), while categorical variables are presented as the number and percentage. Continuous variables were analyzed with the Student's *t*-test. A two-sided probability (*p*) value < 0.05 was considered statistically significant.

## 3. Results

### 3.1. Clinical Characteristics

Tissue specimens collected from 35 patients from November 1, 2018, to January 31, 2020, were positive for *M*. *marinum* infection. The patient characteristics are summarized in Table 1. All 35 patients were ethnic Chinese and included 6 (17.1%) males and 29 (82.9%) females with a mean age of 54.2 (median, 55; IQR, 49–59) years. Of these 35 patients, 19 (54.3%) had a history of a puncture wound from a fish bone, and 13 (37.1%) were employed in the fishing industry or other aquatic-related jobs. The median time from disease onset to confirmation was 3 (IQR, 2.0–4.0) months. None of the patients developed lung abnormalities, as confirmed by chest X-rays, or received immunosuppressant therapy.

**Figure 1:**
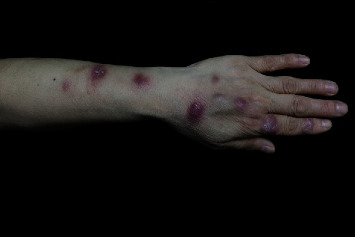
Nodules on fingers and back of hand in patient with *M. marinum* skin infection.

Of these 35 patients, 10 (28.6%) presented with localized skin lesions, and 25 (71.4%) presented with a sporotrichoid spreading pattern. Most of the cutaneous lesions were located on the upper extremities (34/35, 97.1%), especially the fingers and hands (82.9%). Lesions of the hand occurred on the right side in 18 (51.4%) patients, the left side in 12 (34.3%), and bilaterally in 5 (14.3%). Nodules were the most common cutaneous manifestation (27/35, 77.1%). Of these 27 cases, 25 (92.6%) had sporotrichoid patterns (Figure 1). The other skin lesions included erythema (57.1%), papules (37.1%), scales (11.4%), and abscesses (2.9%).

**Figure 2:**
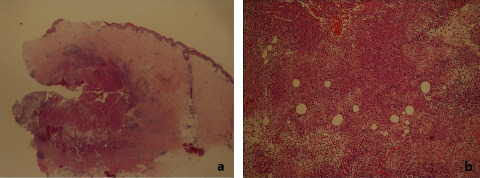
Epitheloid cells forming nonnecrotizing sarcoid-like granuloma (hematoxylin-eosin stain; original magnification: (a) 40x and (b) 100x.

Histopathology of all samples showed granulomatous inflammation (Figure 2). The proportion of 6 positive samples in 35 total samples is 17.1% (95% CI: 6.6%–33.6%) for AFB staining. For PCR-based sequencing of 16S rRNA, the proportion of positive samples is 74.3% (95% CI: 56.7%–87.5%) out of 35 tissue biopsy specimens. In addition, qPCR analysis showed that 17 out of 35 specimens (48.6% [95% CI: 31.4%–66.0%]) were positive.

### 3.2. Treatment and Outcome

**Figure 3:**
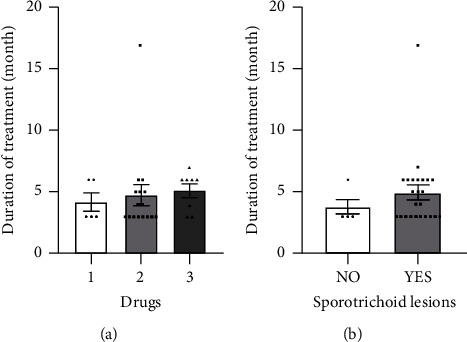
Factors affecting treatment outcomes in *M*. *marinum* infections. (a) Correlation between number of drugs and duration of treatment, *p* > 0.05. (b) Correlation between sporotrichoid lesions and duration of treatment, *p* > 0.05.

All patients received antibiotics. The choice of antibiotics was based on *in vitro* susceptibility testing, which showed that all *M. marinum* strains were sensitive to rifampin, clarithromycin, rifabutin, minocycline, linezolid, and amikacin. All patients were treated with monotherapy or combination therapy of up to 3 antibiotics. Of the 35 patients, 9 (25.7%) received monotherapy with minocycline (6, 17.1%), clarithromycin (2, 5.7%), or doxycycline (1, 2.9%); 17 (48.6%) received dual therapy with clarithromycin and rifampin (13, 37.1%) or clarithromycin and minocycline (4, 11.4%); and 9 (25.7%) received triple therapy with clarithromycin, rifampin, and doxycycline (8, 22.9%) or clarithromycin, rifampin, and ethambutol (1, 2.9%). The median duration of treatment was 4.0 (IQR, 3.0–6.0) months. Clarithromycin was the most frequently used antibiotic, followed by rifampin, doxycycline, and minocycline. Patients with sporotrichoid lesions or those treated with multiple drugs required a longer duration of treatment (Figure 3), although there was no significant difference (*p* > 0.05).

### 3.3. Results of Cytokines by IHC

**Figure 4:**
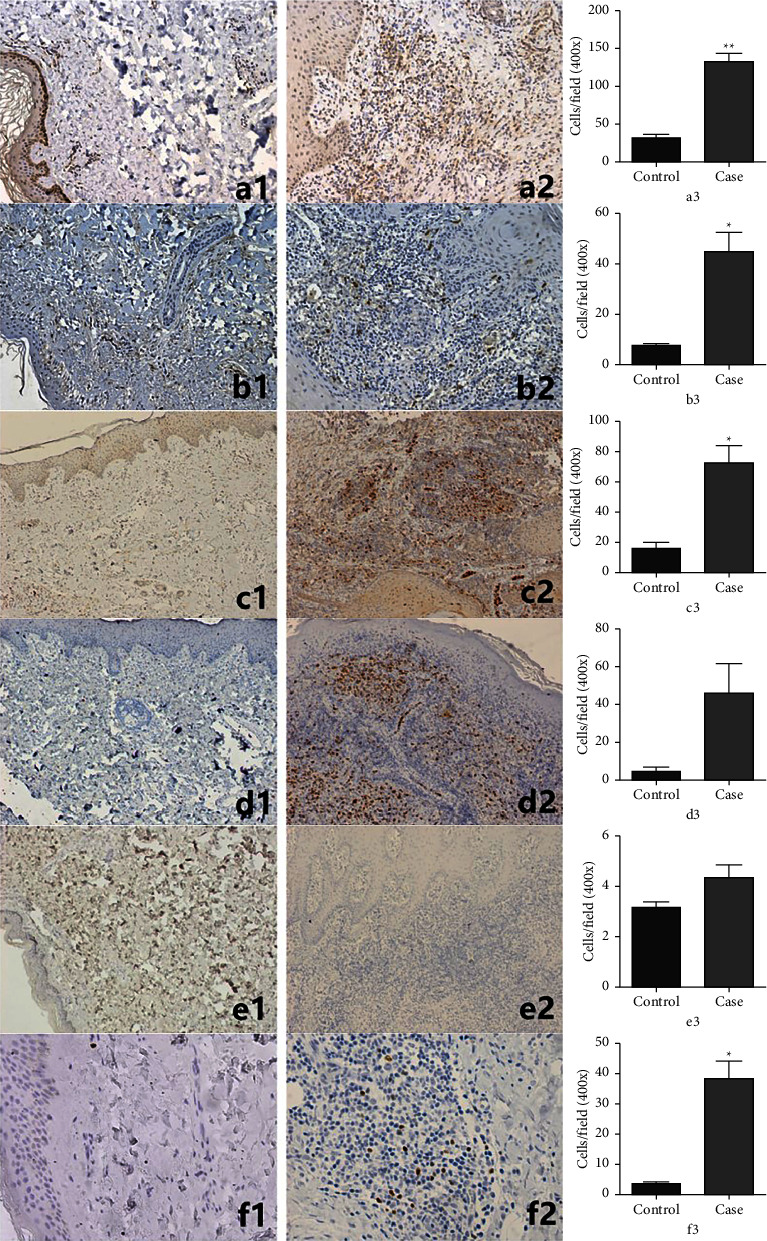
Immunohistochemical staining for detecting IFN-*γ*, IL-4, IL-9, IL-17, IL-22, and FOXP3 in the skin of 9 patients and 5 healthy controls. a1: IFN-*γ* control: original magnification: 200. a2: IFN-*γ* case: original magnification: 200. a3: the expression of IFN-*γ* in *M. marinum* infection, ^*∗∗*^*p* < 0.01. b1: IL-4 control: original magnification: 200. b2: IL-4 case: original magnification: 200. b3: the expression of IL-4 in *M. marinum* infection, ^*∗*^*p* < 0.05. c1: IL-9 control: original magnification: 100. c2: IL-9 case: original magnification: 100. c3: the expression of IL-9 in *M. marinum* infection, ^*∗*^*p* < 0.05. d1: IL-17 control: original magnification: 100. d2: IL-17 case: original magnification: 100. d3: the expression of IL-17 in *M. marinum* infection, *p* > 0.05. e1: IL-22 control: original magnification: 100. e2: IL-22 case: original magnification: 100. e3: the expression of IL-22 in *M. marinum* infection, *p* > 0.05. f1: FOXP3 control: original magnification: 400. f2: FOXP3 case: original magnification: 400. f3: the expression of FOXP3 in *M. marinum* infection, ^*∗*^*p* < 0.05.

IHC analysis was performed using specimens collected from 9 patients and 5 healthy controls to clarify the involvement of cytokines during cutaneous *M. marinum* infection. The expression levels of FOXP3 (*p* < 0.05), IL-9 (*p* < 0.05), IFN-*γ* (*p* < 0.01), and IL-4 (*p* < 0.05) were significantly increased in all lesions of *M. marinum* infection as compared to the normal skin specimens, while there was no significant difference in the expression levels of IL-22 and IL-17 between the patient and control specimens (Figure 4)

## 4. Discussion

In the present study, the clinical features, histopathologic and laboratory data, treatment regimens, and clinical outcomes of 35 cases of *M. marinum* infection were systematically reviewed. In addition, the molecular mechanism underlying the regulation of cytokines involved in *M. marinum* infection was explored.

As one of the most frequently identified NTM species, *M. marinum* causes disease in fish and humans. Human *M*. *marinum* infections are normally caused by exposure of skin wounds to contaminated water of fish tanks or contaminated fish [2, 5]. Although *M. marinum* infections have been reported worldwide [7, 10–20], the cohort of this study had unique characteristics. First, *M. marinum* infection was more common in women than men (82.9% vs. 17.1%, respectively), while there was an opposite trend in previous studies [5, 7, 12, 13, 15, 19, 20]. As a possible explanation for this discrepancy, the cohort of the present study consisted only of ethnic Chinese and women who mainly prepare meals in Chinese society. In addition, 19 cases were caused by puncture wounds from fish bones. Second, the lesions of 25 (71.4%) of the 35 patients had sporotrichoid patterns, slightly higher than that reported in prior studies (5.6%–58.6%) [5, 7, 12, 16, 20]. However, the median age of patients in this cohort was similar to that in previous studies (55 vs. 26–66 years, respectively) [5, 7, 12, 13, 15, 16, 18, 19].

Diagnosis of *M. marinum* infection remains challenging due to the failure to record histories of aquatic exposure, unusual clinical manifestations, and empiric antibiotic treatment prior to culture. In this study, only 19 (54.3%) cases were associated with puncture wounds from fish bones, while the exposure histories of the remaining 16 cases were unknown. Moreover, in clinical practice, skin infection of *M. marinum* is easily confused with sporotrichosis. These factors largely account for the difficulty with diagnosis.

All patients in this study had pathologically confirmed granulomas and 17.1% were positive by AFB staining, similar to previous studies (0%–39%) [7, 10–20]. Although mycobacterial culture is considered the gold standard for diagnosis, sensitivity varies from 41% to 100% [7, 10–20]. Hence, mycobacterial culture is insufficient for the diagnosis of *M*. *marinum* infection, especially for patients receiving antibiotics.

Species-specific PCR has been recently applied in clinical practice for the detection of *M. marinum* infection. In previous studies, PCR was performed to confirm a diagnosis of *M*. *marinum* infection in 34.8%–100% of cases [10, 18–20]. In the present case series, diagnoses of all 35 patients were confirmed either by culture (26 positive, 74.3%) or qPCR analysis (17 positive, 48.6%), indicating that culture combined with qPCR analysis is more accurate for the identification of *M. marinum* infection.

At present, there is no consensus on a standard therapeutic intervention for *M. marinum* infection, as most cases are treated with monotherapy or combination therapies of rifampin, ethambutol, and/or clarithromycin. Notably, multidrug therapy is recommended for invasive infections [6]. Empirical treatment of sporotrichoid lesions is most commonly a combination of 2 or 3 antibiotics with a relatively longer duration of treatment, as reflected in this study and previous reports [12, 15, 21]. Dissemination of *M*. *marinum* infection is rare, and the majority of such cases occur in immunocompromised patients treated with corticosteroids or immunosuppressive therapies or presenting with acquired immunodeficiency syndrome [22]. In this study, 29 (82.9%) of the 35 patients recovered after treatment, similar to the outcomes in previous reports of 68%–100% [7, 10–20]. In addition, the median duration of treatment for cutaneous infection in this study was 4 months, as compared to 2.7–8.3 months in prior case series [7, 10–20].

To the best of our knowledge, no systematic study has investigated the involvement of cytokines in the acquired immune response to *M. marinum* infection. In this study, the expression levels of IFN-*γ*, L-4, IL-9, and FOXP3 were significantly increased in patients with *M. marinum* infection. IFN-*γ* reportedly plays a protective role in the early stages of infection by contributing to the host immune response against pathogens [23]. Moreover, the frequency of NTM infection is reportedly increased by IFN-*γ* signaling and transduction deficiencies [24]. Meanwhile, FOXP3 impairs the immune response against bacilli, thereby facilitating replication [25]. Activation of IL-4 enhances antimycobacterial responses, leading to increased production of inflammatory cytokines upon subsequent exposure. Interestingly, even though IL-4 polarizes macrophages towards the M2 phenotype, which typically promotes mycobacterial growth, an enhanced immune response still persists [26]. However, IL-9 has the ability to mitigate the detrimental impact of IL-4 on the development of cytotoxic T lymphocytes induced by *M. leprae* [27]. IL-4 and IL-9 produced by immune cells are both positively and negatively regulated in response to *M*. *marinum* infection [25].

Notably, there were no significant differences in the expression levels of IL-17 and IL-22 between the patient and control groups in this study, possibly due to the limited number of specimens, whereas previous studies reported that IL-17 and IL-22 acted to inhibit the progression of cutaneous *Mycobacterium* infections [23, 28]. Hence, additional studies are needed to clarify these discrepancies. These results suggest that *M. marinum* infection may cause complex immune responses involving multiple T cell subsets such as Th1, Th2, Th9, Treg, Th17, and Th22.

## 5. Conclusion

Here, the clinical characteristics, diagnoses, and treatment regimens of 35 cases of *M. marinum* infection were systematically reviewed, and the involvement of various cytokines was investigated. Timely and accurate diagnosis and treatment are crucial for successful treatment of cutaneous infections caused by *M*. *marinum*, but they can be particularly challenging in primary hospitals. The duration of patients with sporotrichoid lesions or multidrug treatment was longer, consistent with previous studies, and deserves further attention. An inherent limitation to the present study was the small sample size. Therefore, additional studies with larger sample sizes are needed to further investigate the roles and regulatory mechanisms of cytokines in *M. marinum* infection.

## Figures and Tables

**Table 1:** The characteristics of patients with *M. marinum* infection.

Characteristics	Total (*N* = 35)
Demographic characteristics	
Median age (IQR)	55 (49–59)
Female, no. (%)	29 (82.9%)
Chinese ethnicity, no. (%)	35 (100%)
Aquatic injury, no. (%)	19 (54.3%)
Occupation related to aquatic exposure, no. (%)	13 (37.1%)
Clinical features	
Site of lesion	
Involved fingers/hands	29 (82.9%)
Upper extremities	34 (97.1%)
Left	12 (34.3%)
Right	18 (51.4%)
Bilateral	5 (14.3%)
Multiple skin lesions, no. (%)	25 (71.4%)
Type of lesion	
Nodules, no. (%)	27 (77.1%)
Sporotrichoid spread/nodules (%)	25/27 (92.6%)
Erythemas, no. (%)	20 (57.1%)
Papules, no. (%)	13 (37.1%)
Scales, no. (%)	4 (11.4%)
Abscesses, no. (%)	1 (2.9%)
Diagnosis methods	
In vitro culture positive, no. (%)	26 (74.3%)
qPCR positive, no. (%)	17 (48.6%)
AFB stain positive, no. (%)	6 (17.1%)
Granulomatous inflammation, no. (%)	35 (100%)
Months to diagnosis, median (IQR)^⋀^	3.0 (2.0–4.0)
Treatment	
Antibiotic regimen^*∗*^	
1 drug, no. (%)	9 (25.7%)
2 drugs, no. (%)	17 (48.6%)
3 drugs, no. (%)	9 (25.7%)
Antibiotic agent used^*∗*^	
Clarithromycin, no. (%)	28 (80.0%)
Rifampin, no. (%)	22 (62.9%)
Minocycline, no. (%)	10 (28.6%)
Doxycycline, no. (%)	9 (25.7%)
Ethambutol, no. (%)	1 (2.9%)
Total duration in months, median (IQR)^#^	4.0 (3.0–6.0)
Outcome	
Cure, no. (%)	29 (82.9%)
Lost to follow-up, no. (%)	6 (17.1%)

IQR: interquartile range; ^⋀^one case had unknown onset time; ^*∗*^one case had unknown antibiotic regimens and antibiotic duration; ^#^6 cases had unknown total duration in months.

## Data Availability

The data that support the findings of this study are available from the corresponding author on reasonable request.

## Supplementary Materials

Supplementary Materials Supplementary Table 1. Primer and probe sequences used for qPCR. Supplementary Table 2. Primer sequences used for Sanger sequencing.
