# Supplementary material for: A Series of 35 Cutaneous Infections Caused by Mycobacterium marinum in Han Chinese Population
Source: J Trop Med. 2023 Aug 16;2023:5514275. doi: 10.1155/2023/5514275 (PMC11390208; doi:10.1155/2023/5514275)
Supplement: Supplementary Materials — Supplementary Table 1. Primer and probe sequences used for qPCR. Supplementary Table 2. Primer sequences used for Sanger sequencing. [file 5514275.f1.docx]

**Supplementary Table**

**Supplementary Table 1.** Primer and probe sequences used for qPCR

|  | **Primer pairs 5'-3**' |
| --- | --- |
| ***M. marinum*** | F: CCGATGCCGATCTTGACTTC |
|  | R: AGGTCGTGCCAGTCGTTGTC |
| ***M. haemophilum*** | F: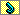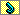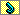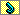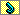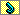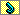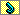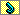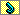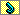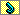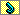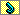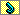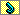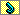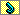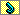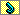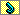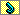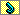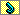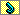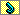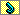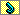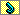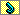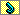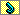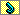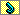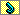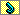 GAGAGCCGGGTGCACAAC |
|  | R: TTGCCTCAGGACCCAACAG |
| ***M. fortuitum*** | F: CCTGTAGTGGGCACGGTTTG |
|  | R: CCAATAGTGTGTCTGGCAGTCAA |
| ***M. leprae*** | F: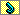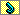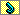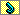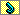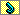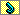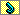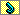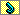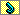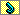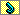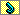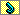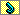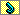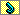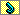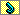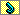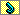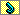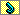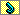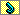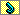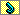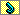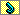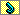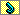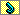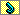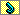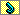 GTGCACGGCAGCAACCTAA |
|  | R: TCGGCATTGAGGTTGAACAC |
| ***M. marinum-probe*** | FAM-CTTCGGTGGACCGCTGA-MGB |
| ***M. haemophilum-probe*** | FAM-CAAATGAATCGCCAGACAC-MGB |
| ***M. fortuitum-probe*** | FAM-TGCACAACAAACTTT-MGB |
| ***M. leprae-probe*** | FAM-TTCCAGGACGCCTACAACGGTGCT-MGB |

**Supplementary Table 2.** Primer sequences used for Sanger sequencing.

|  | **Primer pairs 5'-3**' |
| --- | --- |
| **ITS** | F: TCCGTAGGTGAACCTGCGG |
|  | R: TCCTCCGCTTATTGATATGC |
| **27-F** | F: AGAGTTTGATCMTGGCTCAG |
| **1492-R** | R: TACGGYTACCTTGTTACGACTT |
| **rpoB** | F: TCAAGGAGAAGCGCTACGA |
|  | R: ATGTTGATCAGGGTCTGC |
